# Head-to-head comparison of FNA cytology vs. calcitonin measurement in FNA washout fluids (FNA-CT) to diagnose medullary thyroid carcinoma. A systematic review and meta-analysis

**DOI:** 10.1007/s12020-021-02892-x

**Published:** 2021-10-04

**Authors:** Pierpaolo Trimboli, Jacopo Giannelli, Bernardo Marques, Arnoldo Piccardo, Anna Crescenzi, Maurilio Deandrea

**Affiliations:** 1 Servizio di Endocrinologia e Diabetologia, Ospedale Regionale di Lugano, Ente Ospedaliero Cantonale (EOC), Lugano, Switzerland; 2 Facoltà di Scienze Biomediche, Università della Svizzera Italiana (USI), Lugano, Switzerland; 3 Division of Endocrinology, Diabetes and Metabolism, Department of Medical Science, University of Turin, Turin, Italy; 4 Endocrinology Department, Hospital de Egas Moniz, Centro Hospitalar de Lisboa Ocidental, EPE, Lisbon, Portugal; 5 Nuclear Medicine Department, Ente Ospedaliero “Ospedali Galliera”, Genoa, Italy; 6 Department of Pathology, University Hospital Campus Bio-Medico, Rome, Italy; 7 UO Endocrinologia, Diabetologia e Malattie del metabolismo, AO Ordine Mauriziano Torino, Torino, Italy

**Keywords:** Medullary thyroid cancer, Cytology, Calcitonin, FNA, Washout, Meta-analysis

## Abstract

**Purpose:**

The sensitivity of cytology after fine needle aspiration (FNA-cytology) in detecting medullary thyroid carcinoma (MTC) is low. To overcome this problem, measuring calcitonin (CT) in washout fluid of FNA (FNA-CT) has been largely diffused and showed good performance. However, no evidence-based study exists comparing systematically the sensitivity of FNA-cytology and FNA-CT. This study aimed to systematically review the literature and collect data allowing a head-to-head comparison meta-analysis between FNA-cytology and FNA-CT in detecting MTC lesions.

**Methods:**

The online databases of PubMed/MEDLINE and Scopus were searched until June 2021. Original articles reporting the use of both FNA-cytology and FNA-CT in the same series of histologically proven MTC lesions were included They were extracted general features of each study, number of MTC lesions (nodule and neck lymph nodes), and true positive and false negatives of both FNA-cytology and FNA-CT.

**Results:**

Six studies were included. The sensitivity of FNA-cytology varied from 20% to 86% with a pooled value of 54% (95% CI 35–73%) and significant heterogeneity. The sensitivity of FNA-CT was higher than 95% in almost all studies with a pooled value of 98% (95% CI 96–100%) without heterogeneity. The sensitivity of FNA-CT was significantly higher than that of FNA-cytology.

**Conclusions:**

FNA-CT is significantly more sensitive than FNA-cytology in detecting MTC. Accordingly, FNA-CT represents the standard method to use in patients with suspicious MTC lesions, combined with cytology.

## Introduction

Medullary thyroid carcinoma (MTC) is an infrequent thyroid malignancy originating from C-cells and occurring as a familial disorder in about one in five cases [1, 2]. The timing of the detection of MTC has an impact on patient’s outcome, being a delay in the diagnosis and/or incomplete initial treatment correlated with poorer prognosis [3–5]. Even if the cytological evaluation after fine needle aspiration (FNA-cytology) is recognized as the most reliable tool for the assessment of thyroid nodules, its sensitivity in detecting MTC is concerning [6, 7]. In this context, the measurement of calcitonin (CT), the most sensitive circulating marker of MTC, can be interfered by several factors and its routine use in all patients with nodular thyroid disease has not been generally accepted [1, 2]. On the other hand, CT measurement in the washout fluid of FNA (FNA-CT) has been largely diffused and is recommended by the 2015 American Thyroid Association (ATA) guidelines [2]. Several original papers have been published on this topic but no evidence-based study exists comparing systematically the sensitivity of FNA-cytology and FNA-CT.

In addition to the above technical issues, the need for systematic reviews of diagnostic accuracy has gained interest in the modern cytopathology [8]. In fact, while a lot of original papers reporting several heterogeneous cytological procedures and ancillary techniques are daily published, no strong recommendations can be developed since the absence of evidence-based studies. Then, the precision of the diagnostic estimation in the field of cytopathology can be significantly improved by combining data from different studies, as usually in a meta-analysis.

The present systematic review was conceived to collect data allowing a head-to-head comparison meta-analysis between FNA-cytology and FNA-CT in detecting MTC lesions. Then, an online search strategy was designed to find original papers reporting the performance of both methods in the same patients’ series.

## Materials and methods

### Review conduction

The systematic review was conducted according to the PRISMA-DTA statement [9].

### Search strategy

The online databases of PubMed/MEDLINE and Scopus were searched. Initially, the search was based on the combinations of the following terms (i.e., medullary, thyroid, cytology, FNA, FNAB, FNAC, fine needle aspiration, washout, fluids, and measurement). Later, a specific algorithm, (medullary thyroid OR calcitonin) AND (biopsy OR FNA OR FNAC) AND (washout OR fluid), was used. No beginning date limit was used. No language restriction was adopted. The last search was done on June 28th, 2021. To identify additional studies, the references list of the retrieved articles was screened.

### Study selection

In accordance to the study’s aim, the ideal study to be included was that reporting the use of both FNA-cytology and FNA-CT in the same series of nodules or neck lymph nodes. In fact, only this kind of study could allow us to calculate the pooled sensitivity of both FNAs and compare them in a head-to-head fashion. After screening the online databases, the main exclusion criteria were: (a) articles not within the field of interest of the study; (b) review articles, editorials, letters, comments; (c) articles that did not provide clear study characteristics or reporting overlapping patient data; (d) studies including <10 MTCs. Two researchers (PT, JG) independently reviewed title and abstract of the retrieved articles, applied the above selection criteria, and reviewed the full-text of the remaining articles to determine their inclusion. Disagreements between them were resolved in a mutual consensus.

### Data extraction

For each included study, the following data were extracted: (a) authors and country of origin; (b) journal; (c) year of publication; (d) number of patients evaluated; (e) number of histologically proved MTC lesions (nodule and neck lymph nodes); (f) number of MTC lesions with suspicious or positive report at FNA-cytology; (g) number of MTC lesions with positive FNA-CT; (h) number of MTC lesions with negative FNA results. Then, true positives (TP) and false negatives (FN) of both FNA-cytology and FNA-CT were obtained. In addition, other technical data about the CT measurement were extracted for each study.

### Study quality assessment

The risk of bias of the studies included was assessed independently by two reviewers (PT, JG), according to QUADAS-2 [10]. The risk of bias was rated as low, high, or unclear.

### Statistical analysis

The sensitivity of FNA-cytology and FNA-CT in detecting MTC lesions was calculated from each article on per lesion-based analysis by using this formula: Sensitivity = TP / (TP + FN). FNA-cytology was considered as TP when an MTC lesion was defined as malignant or when it was defined suspicious for MTC by authors of the studies. FNA-CT was considered as TP according to the criterion adopted by the authors of the studies. A random effects model was used. Pooled data were presented with 95% confidence intervals (95% CI). To compare the sensitivity between FNA-cytology and FNA-CT, a significant difference could be detected if the 95% CIs of the two FNAs were not overlapping. Heterogeneity was assessed by I_2_ index and it was significant with value >50%. Statistical analyses were performed by using OpenMeta[Analyst] (open-source software developed by the Center for Evidence Synthesis in Health, Brown University, Providence, RI, USA).

## Results

### Articles retrieved

**Fig. 1:**
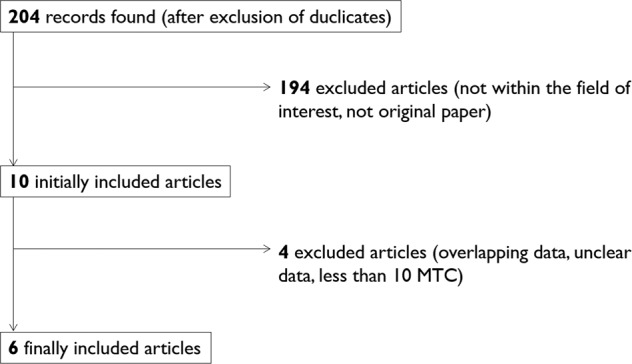
Flow of search of articles

According to our searching algorithm, a number of 204 records was retrieved. After excluding duplicates and applying the above selection criteria, 6 articles [11–16] were finally included in the present systematic review (Fig. 1). The main characteristics of these studies are detailed in Table 1.

**Table 1:** Main features of the 6 included articles

First author	Year	Country	Journal	Study design	Patients (*n*)	Lesions undergone FNA	
						Nodules (*n*)	Lymph nodes (*n*)
Boi	2007	Italy	J Clin Endocrinol Metab	Retrospective, single-center	23	18	18
Diazzi	2013	Italy	Endocr Pract	Prospective, single-center	27	60	0
Trimboli	2014	Italy	Clin Endocrinol	Retrospective, multi-center	88	82	3
De Crea	2014	Italy	Arch Otoryngol Ital	Prospective, single-center	38	NA	NA
Marques	2020	Portugal	Int J Endocrinol	Retrospective, single-center	42	0	69
Liu	2021	China	Endocrine	Prospective, single-center	78	87/84^a^	0

^a^87 nodules underwent FNA-CT and 84 of them underwent also FNA-cytology

### Qualitative analysis (systematic review)

From the six studies included in the systematic review, four were published by Italian authors and the remaining two were published by researchers from Portugal and China. One study presented a multi-center series while the other studies were single-center. All studies included patients with suspicious MTC or recurrent MTC based on high serum CT levels. The overall number of patients enrolled in the studies was 278 and the lesions that undergone FNA-cytology and FNA-CT was 399. Finally, a total of 173 histologically proven MTC lesions were available. The sensitivity of both FNA-cytology and FNA-CT was reported in all six studies. In addition, some of them [14, 15] also calculated the sensitivity of FNA-CT/serum CT ratio. Table 2 illustrates the FNA results recorded in the six studies. The technical approach used in the six studies to prepare samples for FNA-cytology and FNA-CT is summarized in the Table 3.

**Table 2:** Histologically proven MTC lesions and number of positive cases among them

First author	Year	MTC lesions at histology	Positive/Suspicious FNA-cytology	Positive FNA-CT	Positive FNA-CT/serum CT ratio
Boi	2007	21	13	21	NR
Diazzi	2013	10	1	10	NR
Trimboli	2014	34	20	34	NR
De Crea	2014	18	9	16	15
Marques	2020	21	18	21	7
Liu	2021	69	39	68	NR

*NR* not reported/performed

**Table 3:** Management of FNA samples for cytology and FNA-CT in the 6 studies

First author	Year	FNA-cytology	FNA-calcitonin		
		Preparation	Sample dilution	CT assay	FNA-CT cut-off proposed (pg/ml)
Boi	2007	Conventional smears	0.5 ml CT-free buffer	Immulite	36 (three times the high serum CT in non-MTC cases)
Diazzi	2013	Conventional smears plus ICC	1.0 ml saline	Dia Sorin (CLIA)	17 (after multiple cut-offs comparison)
Trimboli	2014	Conventional smears plus ICC	1.0 ml saline	Immulite	39.6 (serum CT cut-off obtained by ROC analysis plus interlaboratory CV)
De Crea	2014	Thin-layer and conventional smears plus ICC	0.5 ml saline	Dia Sorin (CLIA)	10.4 (ROC analysis)
Marques	2020	Conventional smears	1.0 ml saline	Siemens Healthcare Diagnostics (CLIA)	23 (ROC analysis)
Liu	2021	Conventional smears	1.0 ml saline	Mindray Medical International (CLIA)	36 (according to Boi et al. [11])

*ICC* immunocytochemistry, *CV* coefficient of variation

### Qualitative assessment

The risk of bias was assessed based on four study characteristics (Table 4). The risk of selection bias was unclear in four studies, low in one and high in another one. Remarkably, the standard of reference (i.e., surgical and histological findings) was the optimal one to evaluate the sensitivity of FNAs in all cases.

**Table 4:** Quality assessment of the studies included in the meta-analysis

		Risk of bias				Feasibility		
First author	Year	Patient selection	Study test	Reference standard	Timing	Patient selection	Study test	Reference standard
Boi	2007	U	L	L	L	L	L	L
Diazzi	2013	U	L	L	L	L	L	L
Trimboli	2014	U	L	L	L	L	L	L
De Crea	2014	H	L	L	L	L	L	L
Marques	2020	U	L	L	L	L	L	L
Liu	2021	L	L	L	L	L	L	L

*H* high, *L* low, *U* unclear

### Quantitative analysis (meta-analysis)

**Fig. 2:**
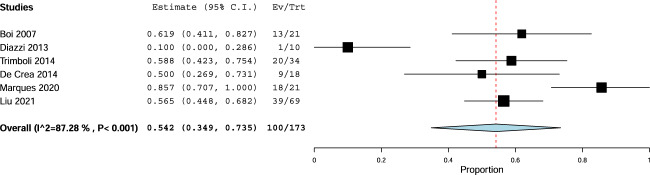
Pooled sensitivity of FNA-cytology. Diamond indicates the pooled sensitivity. Square size indicates the sample. Line indicates 95% CI

The sensitivity of FNA-cytology recorded in the six studies varied from 20% to 86%. The pooled sensitivity of FNA-cytology obtained by the meta-analysis was 54% with 95% CI from 35 to 73% and significant heterogeneity (Fig. 2). To explore the heterogeneity both study design and date of publication were considered as influencing factors. The FNA-cytology sensitivity was 69% (95% CI 51–87%) in the subgroup of retrospective studies [11, 13, 15] and 39% (95% CI 9–68%) in the subgroup of prospective ones [12, 14, 16]; also, it was 70% (95% CI 42–99%) in the subgroup of most recent studies [15, 16] and 45% (95% CI 20–69%) in the subgroup of the previous ones [11–14].

**Fig. 3:**
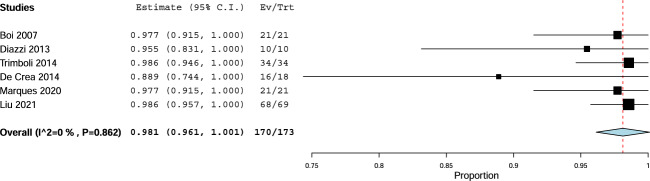
Pooled sensitivity of FNA-CT. Diamond indicates the pooled sensitivity. Square size indicates the sample. Line indicates 95% CI

The sensitivity of FNA-CT was higher than 95% in all studies except for one that recorded 88%. The pooled sensitivity of FNA-CT was 98 with 95% CI from 96% to 100% in the complete absence of heterogeneity (Fig. 3).

Since the 95% CI of FNA-cytology and FNA-CT were not overlapping, the pooled sensitivity of FNA-CT could be assessed as significantly higher than that observed for FNA-cytology.

## Discussion

The diagnosis of MTC can be challenging and the significant limitations of FNA-cytology in detecting this neoplasm are recognized. As reported in a meta-analysis published in 2015 [6], only 56% of histologically proven MTC lesions can be correctly detected by cytological evaluation. The results of that meta-analysis were not particularly different compared to single studies enrolling large or small series of MTC. Elisei et al. [17] found a 45% sensitivity in 44 MTCs, Bugalho et al. [18] recorded a 63% sensitivity in 67 cases, and Essig et al. [19] collected 245 MTCs from 12 institutions over 29 years and 112 (45.7%) were correctly diagnosed. In this context, two historical studies with different design must be mentioned [20, 21], as they found a positive predictive value regarding FNA-cytology of 89% [20] and 81% [21], respectively. The present meta-analysis found FNA-cytology to have a 54% sensitivity, thus perfectly confirming the previous results obtained in a different collection of studies [6]. In recent years, the use of FNA-CT in patients with suspicious MTC (i.e., high serum CT levels) has gained momentum and this strategy was introduced in 2015 ATA guidelines [2]. Since its first description [11], FNA-CT showed high sensitivity and an apparently higher diagnostic performance than FNA-cytology. The main advantage of FNA-CT is that it can be used in any institution with a laboratory department. Even if the conventional assays for CT are not developed for this use, no specific concerns regarding the measurement of CT in FNA fluids have been reported in the literature. The major potential limitation of FNA-CT should be the lack of a fixed threshold to be adopted in the clinical practice [22]. Nevertheless, a three-point discussion has to be addressed about this debated issue: (1) as intrinsically showed in the data of the herein included studies, adopting an institutional cut-off can solve this problem; (2) the complete absence of heterogeneity we observed in the pooled sensitivity of FNA-CT could intrinsically demonstrate that the different technical approaches used to prepare and measure FNA-CT (Table 3) did not influence the final result; (3) the FNA-CT values recorded in MTC lesions are generally very high enabling us to correctly diagnose it. Other potential weaknesses of FNA-CT are fully shared with alternative diagnostic options (i.e., immunocytochemistry for CT). Mainly, C cell hyperplasia may result as a false positive case for both FNA-CT and immunocytochemistry [22]. However, while immunocytochemistry cannot be performed in a suboptimal sample collected by FNA, FNA-CT can be perfectly evaluated in these cases. All in all, it has to be underlined that we need a high value of serum CT before FNA to better select the cases in which the use of FNA-CT might be appropriate, being this clinical strategy debatable [23]. Moreover, measuring serum CT value represents a key-information to better enhance the morphological evaluation of FNA specimens. In order to improve the performance of both FNA-CT and FNA-cytology, testing for serum CT in patients undergoing FNA should be considered in clinical setting. The present meta-analysis demonstrates with strong statistical power that the sensitivity of FNA-CT is significantly higher than that of FNA-cytology. The use of FNA-CT has been recommended by ATA guidelines with Grade B recommendation [2]. With the present new evidence-based data, the use of FNA-CT should be upgraded to Grade A recommendation (strong evidence) in the future revised guidelines. In addition, the use of FNA-CT should be included in next revised guidelines for thyroid cytology (i.e., Bethesda system, etc.).

**Fig. 4:**
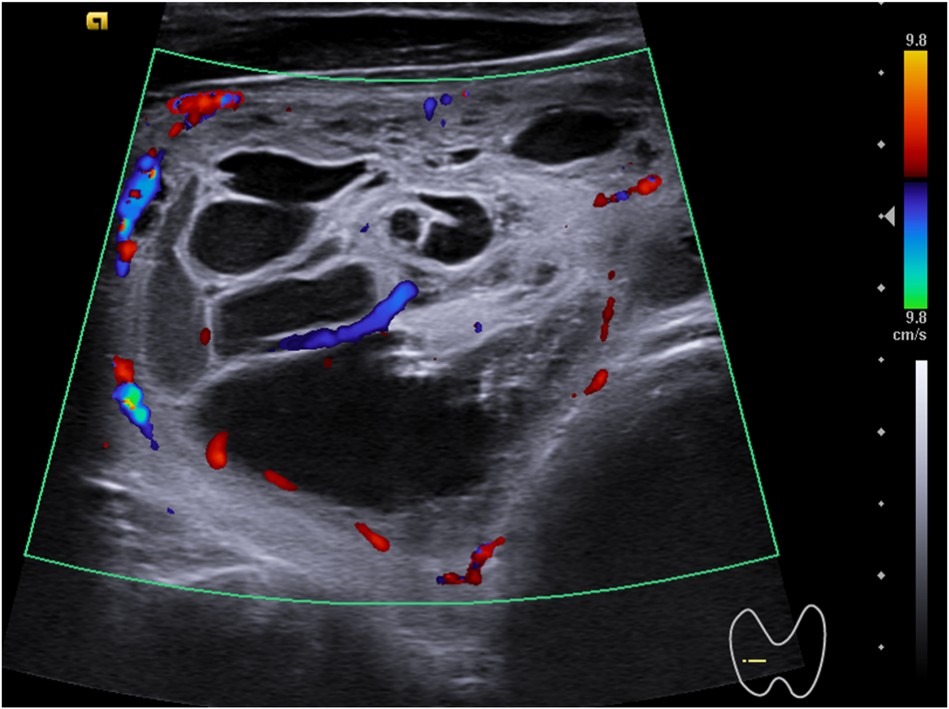
Ultrasound presentation of one case of MTC. The nodule is placed in the right thyroid lobe and presents as mixed with isoechoic and anechoic parts, mildly vascularized, with largest diameter of 44 mm.

A critical discussion about the herein recorded results should be addressed. As showed by our sub-analysis, the pooled sensitivity of FNA-cytology was negatively influenced by its poor performance in prospective studies; this corroborates that the cytological diagnosis of MTC during the clinical practice is more difficult than its detection when reviewing the cytological specimens for research purpose. Furthermore, the FNA-cytology sensitivity of the most recent studies [15, 16] was improved with respect that of the previously published ones; we can speculate that cytopathologists have recently become more aware of this difficult diagnosis and, maybe, have been positively influenced by the concomitant use of FNA-CT. In addition, one study [12] showed a very poor sensitivity of cytology; this finding might be due to several reasons such as the prospective enrollment of cases and the potential difficult sampling in small nodules (i.e., median size 9.5 mm). Furthermore, it should be relevant to underline the role of different preparation for cytological evaluation adopted by the six studies (Table 3). Overall, the use of the conventional smears is the most widely used method while immunohistochemistry can be crucial to detect MTC [20–22]. Regarding FNA-CT, the low sensitivity recorded by De Crea et al. [14] might be due to the prospective design and the enrollment of thyroidectomized patients only; some patients of that series might be operated-upon following suspicious serum CT and/or FNA-cytology despite of low CT value in the washout fluids (these cases were described as false-negative FNA-CT cases). Anyway, the heterogeneous ultrasound presentation of MTC has to be taken into account when critically analyzing the limitations of FNA procedure in detecting it. In fact, as recently reported by Matrone et al. [24], <50% of MTCs is correctly identified as suspicious by ultrasound systems for risk stratification of thyroid nodule. The Fig. 4 illustrates the ultrasound presentation of one MTC case.

Due to the aim and conceptualization of the present study, the specificity of FNAs could not be known. However, in most cases, patients with negative FNAs were not operated upon and we had no strong reference of standard to calculate the true negatives and false positives cases with high reliability.

As usual, some potential limitations may affect the present meta-analysis. First, no large-sample studies were found and this influenced the pooled sample size. This obviously depends on the infrequent incidence of MTC. The herein recorded difference in sensitivity between FNA-CT and FNA-cytology was significant (i.e., non-overlapping 95% CIs) and this data suggests to perform, as a further proof, prospective multicenter studies with direct comparison between FNA-CT and FNA-cytology. Second, some studies were retrospective. This could influence the findings of FNA-cytology (with potential over-estimate) and explain the heterogeneity observed only in FNA-cytology. Third, the series of patients should be not consecutive and a potential selection bias should be taken into account. Fourth, we must be aware that a different use of serum CT testing could be adopted by different institutions. This might have influenced in clinical practice the selection of patients undergoing FNA with or without measurement of CT in washout fluids. However, the major strength of the present data is that FNA-CT showed excellent sensitivity with no discordant data among studies (i.e., I_2_ = 0).

In conclusion, our meta-analysis (1) confirms that FNA-cytology can only detect approximately a half of histologically proven MTC lesions, and (2) demonstrates that FNA-CT is significantly more sensitive than FNA-cytology. Accordingly, FNA-CT is the standard method to use in patients with suspicious MTC lesions and it has to be combined with FNA-cytology in all cases.

## Data Availability

The datasets generated during and/or analyzed during the current study are not publicly available but are available from the corresponding author on reasonable request.
